# Coxsackie B virus-induced myocarditis in a patient with a history of lymphoma: A case report and review of literature

**DOI:** 10.1097/MD.0000000000037248

**Published:** 2024-03-08

**Authors:** Qian Zhang, Jia Yuan, Wei Zhao, Weiwei Ouyang, Bowen Chen, Yehong Li, Junling Tao, Xianjun Chen, Guangsu Li, Zhendong Guo, Ying Liu

**Affiliations:** a Department of Critical Care Medicine, The Affiliated Hospital of Guizhou Medical University, Guiyang, China; b Department of Oncology, The Affiliated Hospital of Guizhou Medical University and The Affiliated Cancer Hospital of Guizhou Medical University, Guiyang, China; c Beijing Goldstandard Medicine Independent Clinical Laboratory Co. Ltd., Beijing, China.

**Keywords:** Coxsackie B virus, lymphoma, myocarditis, pathogen-targeted next-generation sequencing

## Abstract

**Introduction:**

In rare occasions, coxsackievirus infections can cause serious illness, such as encephalitis and myocarditis. The immunotherapies of cancer could increase the risk of myocarditis, especially when applying immune checkpoint inhibitors. Herein, we report a rare case of Coxsackie B virus-induced myocarditis in a patient with a history of lymphoma.

**Case presentation:**

A 32-year-old woman was admitted to the hospital with recurrent fever for more than 20 days, and she had a history of lymphoma. Before admission, the positron emission tomography/computed tomography result indicated that the patient had no tumor progression, and she was not considered the cancer-related fever upon arriving at our hospital. Patient’s red blood cell, platelet count, and blood pressure were decreased. In addition, she had sinus bradycardia and 3 branch blocks, which was consistent with acute high lateral and anterior wall myocardial infarction. During hospitalization, the patient had recurrent arrhythmia, repeated sweating, poor mentation, dyspnea, and Coxsackie B virus were detected in patient’s blood samples by pathogen-targeted next-generation sequencing. The creatine kinase, creatine kinase MB, and N-terminal pro-brain natriuretic peptide were persistently elevated. Consequently, the patient was diagnosed with viral myocarditis induced by Coxsackie B virus, and treated with acyclovir, gamma globulin combined with methylprednisolone shock therapy, trimetazidine, levosimendan, sildenan, continuous pump pressors with *m*-hydroxylamine, entecavir, adefovir, glutathione, pantoprazole, and low-molecular-weight heparin. Her symptoms worsened and died.

**Conclusion:**

We reported a case with a history of lymphoma presented with fever, myocardial injury, who was ultimately diagnosed with Coxsackie B virus-induced myocarditis. Moreover, pathogen-targeted next-generation sequencing indeed exhibited higher sensitivity compared to mNGS in detecting Coxsackie B virus.

## 1. Introduction

Myocarditis is an immunological response that causes myocardial inflammation as a result of an attack on cardiac tissue by antigens such as viruses, resulting in edema, remodeling, and fibrosis of the heart muscle.^[1]^ Myocarditis may present as acute, fulminant, subacute, or chronic conditions, and acute myocarditis is able to cause acute cardiogenic shock, acute heart failure, and arrhythmia.^[2]^ The typical features of myocarditis comprise chest pain, fever, dyspnea, palpitations syncope, and fatigue.^[3]^ Dilated cardiomyopathy (DCM) is the most common consequence of myocarditis, characterized by dilatation of heart chambers and impairment of systolic function.^[4]^ It has been demonstrated that multiple viruses are correlated with the development of viral myocarditis, such as coxsackievirus, parvovirus, adenovirus, and cytomegalovirus, and coxsackievirus is most common causes of viral myocarditis, especially Coxsackie B virus.^[5]^

Coxsackievirus belongs to the nonenveloped RNA virus of the *Picornaviridae* family. As an enteric virus, coxsackievirus infects the gastrointestinal system before spreading to peripheral organs and causing illness.^[6]^ The majority of coxsackievirus infections are asymptomatic or have moderate upper respiratory tract symptoms. In rare occasions, coxsackievirus infections can cause serious illness, such as encephalitis and myocarditis. Coxsackievirus-induced myocarditis increases the risk of developing persistent DCM, a leading cause of heart failure.^[7]^ Coxsackieviruses can be categorized into 23 serotypes of Coxsackie A virus and 6 serotypes of Coxsackie B virus.^[7,8]^ Coxsackie B virus infections are highly contagious, and it is one of the most prevalent causes of viral myocarditis in children and adolescents, accounting for 10% to 20% of all instances of myocarditis and DCM.^[9,10]^ The next-generation sequencing (NGS), including metagenomic NGS (mNGS) and pathogen-targeted NGS (ptNGS) were developed and has been used to identify pathogens.^[11]^

Furthermore, along with the coxsackievirus, cancer-related immunotherapy, in particular immune checkpoint inhibitors (ICIs), can increase the likelihood of developing myocarditis.^[12]^ For example, 2 individuals with metastatic melanoma who developed myocarditis immediately after receiving nivolumab in combination with ipilimumab therapy.^[13]^ ICIs could target the host negative immunological regulators, causing the immune system to become activated against the patient’s cancer cells,^[14]^ but which may cause the autoimmune or inflammatory response in normal tissue resulting in immune-related adverse events in many organs.^[15,16]^ It has been reported that compared to nivolumab alone the incidence of myocarditis is higher and more severe in patients treated with a combination of ipilimumab and nivolumab.^[13]^ In addition, other types of immunotherapies may have cardiovascular side effects. The usage of genetically engineered T cell receptors against a cancer antigen (MAGE-A3) resulted in myocarditis.^[17]^ However, the myocarditis induced by coxsackievirus in patients with history of cancer has been rarely reported. Here, we report a rare case of Coxsackie B virus-induced myocarditis in a patient with a history of lymphoma.

## 2. Case presentation

A 32-year-old woman was admitted to the hospital with recurrent fever for more than 20 days. The patient had a history of B-cell non-Hodgkin lymphoma and treated with surgical resection and chemotherapy from September 2021 to March 2022. On August 1, 2022, the patient had fever (the maximum body temperature reached 40°C), white blood cell (WBC) count was 2.5 × 10^9^/L and was treated with amoxicillin and leucocytosis. Subsequently, the patient continued to have fever (38.6–38.9°C), accompanied by chills, headache, nausea, and vomiting and was admitted to the Affiliated Cancer Hospital of Guizhou Medical University on August 11th. The body temperature was 40°C, WBC count was 19.96 × 10^9^/L and the proportion of lymphocytes was 5.2%, and she was treated with anti-bacterial infection. During hospitalization, the patient continued to have fever (37.6°C–39.2°C), WBC was 1.88 × 10^9^/L, lymphocytes and neutrophile granulocyte counts were 0.41 × 10^9^/L and 1.12 × 10^9^/L, respectively. The mNGS results showed that no pathogen was detected. The positron emission tomography/computed tomography (PET-CT) showed that that patient had no tumor progression and not considered the cancer-related fever.

On August 25th, the patient was transferred to our hospital, the Affiliated Hospital of Guizhou Medical University. Upon arriving at the hospital, the routine blood test results revealed that the WBC (3.76 × 10^9^/L), neutrophil absolute (2.81 × 10^9^/L), lymphocyte absolute (0.50 × 10^9^/L), red blood cell count (3.58 × 10^12^/L), hemoglobin volume (110.00 g/L), hematocrit (33.60%), and platelet count (76.00 × 10^9^/L) were lower. Table 1 listed the detailed results of renal and liver function, myocardial enzyme, and lymphocyte subsets of patient. Electrocardiogram (ECG) showed that she had sinus bradycardia (HR = 59 bpm) and 3 branch blocks (complete right bundle branch, left anterior branch block, and third degree atrioventricular block), which was consistent with acute high lateral and anterior wall myocardial infarction. A coronary angiogram was then performed and no significant aberrant stenosis was found. Her blood pressure (67/43 mm Hg) was decreased.

**Table 1:** The detailed results of renal and liver function, myocardial enzyme, and lymphocyte subsets of patient.

	Projects	Values	Normal reference
Renal and liver function	Urea (mmol/L)	5.52	2.60–7.50
	Crea (μmol/L)	63	41.00–73.00
	ALT (U/L)	33	7.00–45.00
	AST (U/L)	120.9	13.00–40.00
	TBIL (μmol/L)	4.9	0.00–21.00
	DBIL (μmol/L)	1.8	0.00–4.00
	ALB (g/L)	42.9	40.00–55.00
Myocardial enzyme	Hs-cTnT (ng/mL)	2.23	0.00–0.014
	Myo (ng/mL)	131.2	25.00–58.00
	NT-proBNP (pg/mL)	1876	0.00–125.00
	CK (U/L)	459	50.00–310.00
	CK-MB (U/L)	88.2	0.00–25.00
	LDH (U/L)	756	120.00–250.00
	α-HBDH (U/L)	548	72.00–182.00
	CRP (mg/L)	5.25	0.00–6.00
Lymphocyte subsets	Total T cells (%)	96.90	64.62–77.08
	CD8 + T cells (%)	75.51	24.81–35.99
	CD4 + T cells (%)	20.15	32.69–44.23
	NK cells (%)	2.98	14.47–30.27
	B cells (%)	0.12	6.38–12.46

ALB = albumin, ALT = alanine aminotransferase, AST = aspartate aminotransferase, CK = creatine kinase, CK-MB = creatine kinase MB, Crea = creatinine, CRP = C reactive protein, DBIL = direct bilirubin, Hs-cTnT = human high sensitivity cardiac troponin T, LDH = lactate dehydrogenase, Myo = myoglobin, NK = natural killer cell, NT-proBNP = N-terminal pro-brain natriuretic peptide, TBIL = total bilirubin, α-HBDH = α-hydroxybutyrate dehydrogenase.

On August 26th, the patient was transferred to the ICU for further life-supportive therapy. She exhibited apathetic, repeated sweating, intermittent low-grade fever, and low blood pressure (122/82 mm Hg). The effusion in both lungs was increased and left ventricular contraction was markedly diminished. Anti-nuclear antibody profiles showed that cardiolipin antibodies and anti-neutrophils granulocytes were negative. The anti-rubella virus antibody IgG (7.01 AU/mL), anti-herpes simplex virus type I/II antibody IgG (15.70 AU/mL) and anti-cytomegalovirus antibody IgG were elevated. During the hospitalization from August 26th to August 29th, the myocardial zymography of patient was presented in Table 2. During hospitalization from August 27th to August 29th, the patient had recurrent arrhythmia, repeated sweating, poor mentation, and dyspnea. On August 29th, the patient’s blood samples were collected and subjected to ptNGS, and Coxsackie B virus were detected.

**Table 2:** The myocardial zymography of patient.

Projects	Date				Reference
	August 26th	August 27th	August 28th	August 29th	
Hs-cTnT (ng/mL)	3.03	2.24	3.05	4.65	0.00–0.014
Myo (ng/mL)	122.90	150.50	264.40	427.00	25.00–58.00
NT-proBNP (pg/mL)	9793.00	20,317.00	14,933.00	26,427.00	0.00–125.00
CK (U/L)	649.00	754.00	1403.00	1197.00	50.00–310.00
CK-MB (U/L)	83.52	109.35	322.50	170.68	0.00–25.00
LDH (U/L)	841.00	1197.00	1239.00	1602.00	120.00–250.00
α-HBDH (U/L)	669.00	952.00	1155.00	1341.00	72.00–182.00

CK = creatine kinase, CK-MB = creatine kinase MB, α-HBDH = α-hydroxybutyrate dehydrogenase, Hs-cTnT = human high-sensitivity cardiac troponin T, LDH = lactate dehydrogenase, Myo = myoglobin, NT-proBNP = N-terminal pro-brain natriuretic peptide.

Based on the above results, the patient was finally diagnosed with myocarditis caused by infection of Coxsackie B virus, cardiogenic shock, and pulmonary infection. Then she received the following treatments: acyclovir fighting virus, gamma globulin combined with methylprednisolone shock therapy, trimetazidine nutriting the myocardium, levosimendan and sildenan strengthening the heart, *m*-hydroxylamine continuous pumping, moxifloxacin, entecavir and adefovir fighting infection, glutathione protects liver, pantoprazole suppresses stomach acid, and low-molecular-weight heparin against coagulation. On the evening of August 29th, the patient’s conditions deteriorated, while her family refused extracorporeal membrane oxygenation (ECMO) treatment and decided to give up rescuing. The patient finally died.

## 3. Discussion and conclusion

The majority of coxsackievirus infections are asymptomatic or have moderate upper respiratory tract symptoms, such as mild upper respiratory tract infections, nonspecific febrile illness, and rashes.^[18]^ However, in rare occasions, coxsackievirus infections can cause serious illness, such as encephalitis and myocarditis.^[7]^ The patient described in the current case is a woman with fever recurred for more than 20 days, and she had a history of lymphoma. Before admission, the PET-CT result indicated that the patient had no tumor progression and not considered the cancer-related fever in the Affiliated Cancer Hospital of Guizhou Medical University. Upon arriving at our hospital, the red blood cell and platelet count and blood pressure were decreased of the patient. In addition, she had sinus bradycardia and 3 branch blocks (complete right bundle branch, left anterior branch block, and 3rd degree atrioventricular block), which consistent with acute high lateral and anterior wall myocardial infarction. During hospitalization, the patient had recurrent arrhythmia, repeated sweating, poor mentation, and dyspnea, with Coxsackie B virus being detected in patient’s blood samples by ptNGS. The creatine kinase (CK), creatine kinase MB (CK-MB) and N-terminal pro-brain natriuretic peptide (NT-proBNP) were persistently elevated. Consequently, the patient was diagnosed with viral myocarditis induced by Coxsackie B virus.

It has been reported that the immunotherapies of cancer could increase the risk of myocarditis, especially ICIs.^[12]^ Johnson et al^[13]^ reported that 2 metastatic melanoma patients and developed myocarditis shortly after nivolumab combined with ipilimumab treatment. In this case, patient’s CK, CK-MB, and NT-proBNP were remarkably elevated, which were the biomarkers for myocarditis in cancer patients receiving immunotherapy.^[12]^ Therefore, the development of myocarditis in this patient may be related to her history of lymphoma. These evidences indicated that the cancer patients receiving immunotherapy should be closely monitored for the development of myocarditis and taken the corresponding medication. In general, myocarditis should be considered in cancer patients who have a rise in cardiac troponin, ECG changes, arrhythmia, or abnormalities of left ventricular systolic function, particularly if the symptoms were unexplained by another diagnosis.^[12]^ Noteworthily, in the Affiliated Cancer Hospital of Guizhou Medical University, no pathogens were detected in the patient’s blood samples using mNGS. mNGS gives an impartial method to pathogen detection, allowing for the identification of both known and unknown infections, as well as the discovery of novel species.^[19]^ mNGS has a better sensitivity for pathogen detection and is less impacted by prior antibiotic exposure. However, it is a challenging task for mNGS to define precise microbial profiles that can aid in the diagnosis or prediction of disease development. Compared to traditional assays, ptNGS offers higher sensitivity and specificity in diagnosing bacterial and viral pathogens in the alveolar lavage fluid of patients with severe community-acquired pneumonia.^[20]^ Gao et al^[11]^ reported that compared with mNGS, ptNGS had a higher sensitivity rate, lower economic costs, and a shorter time to identify pathogens in cerebrospinal fluid. In the present case, the Coxsackie B virus in blood samples was detected by ptNGS but not by mNGS. Obviously, ptNGS had advantages of high-detection sensitivity that is unaffected by the human genome or background bacteria, decreased detection cost, reduced sample transportation needs, and quantitative detection of pathogens.

Coxsackie B virus has been found to replicate in the gastrointestinal system and spleen before spreading to the heart and causing damage. In the early stages of infection, fever, diarrhea, nausea, vomiting, exhaustion, and body pains were common symptoms of patients. Myocarditis might occur around 2 weeks after infection, which might manifest as chest discomfort of varying intensity, commonly around the rib–breastbone junction.^[21]^ The treatment of Coxsackie B virus-induced myocarditis included immunosuppressive agents, such as prednisone, cyclosporine, IV immunoglobulin, and antivirals, such as pleconaril, interferon, and acyclovir. Moreover, fever and mild pain could be treated with nonsteroidal anti-inflammatory drugs or acetaminophen.^[22]^ However, there was no pathogen was detected in the patient’s blood at the beginning of her fever by mNGS, which missed the optimal time for treatment. In the present case, patient received the following treatments: acyclovir fighting virus, gamma globulin combined with methylprednisolone shock therapy, trimetazidine nutriting the myocardium, levosimendan and sildenan strengthening the heart, *m*-hydroxylamine continuous pumping, moxifloxacin, entecavir and adefovir fighting infection, glutathione protects liver, and pantoprazole suppress stomach acid. Among which, gamma globulin combined with methylprednisolone pulse therapy and trimetazidine treatments belonged to hormonotherapy. Gamma globulin is extracted from the plasma of healthy individuals and can rapidly increase the body IgG level after intravenous administration, which can directly neutralize toxins and prevent the virus from replicating in the body and assist in the killing of pathogens.^[23,24]^ Methylprednisolone is a synthetic glucocorticoid belonging to the class of intermediate acting hormones that binds to specific receptors within the cytosol and potently modulates immune mechanisms, antagonizes inflammatory delivery.^[25]^ Previous studies have shown that early administration of high-dose glucocorticoids and gamma globulin can effectively improve myocardial function and improve clinical outcomes in patients with severe viral myocarditis.^[26]^ Trimetazidine is a piperazine derivative used as an anti-anginal agent. In heart failure, trimetazidine could improve energy efficiency via a variety of ways (such as increased glucose metabolism by increasing the rate-limiting enzyme activity of glucose aerobic oxidation), it could enhance cardiac function through regulating cardiomyocyte autophagy and play anti-inflammatory effects via modulating the expression of inflammatory factors.^[27]^ Wu et al^[28]^ reported that trimetazidine combined with Chinese herbal injections could significantly reduce the levels of CK, CK-MB, and lactate dehydrogenase. Furthermore, trimetazidine inhibits neutrophil accumulation and infiltration, protects cardiac function, and slows gluconeogenesis, all of which successfully protect the ischemic myocardium.^[29]^ It has been reported that Coxsackie B3 virus could interact with human neutrophils to improve neutrophil survival and promote CD11b expression, cytokine and IL-8 release, and that neutrophils can recognize Coxsackie B3 virus via tlr-8 and activate NF-κB. Whereas, in Coxsackie B3 virus infected mice, the depleted neutrophil could reduce the myocardial inflammation.^[30]^ In this case, after treatment with gamma globulin combined with methylprednisolone pulse methylprednisolone and trimetazidine, the biomarkers of myocarditis, CK and CK-MB levels were reduced. These evidences indicated that hormonotherapy has clinical efficacy in the treatment of Coxsackie B virus-induced myocarditis patients with a history of cancer.

In conclusion, we reported a case with a history of lymphoma presented with fever, myocardial injury, who was ultimately diagnosed with Coxsackie B virus-induced myocarditis and unsuccessfully treated with multidisciplinary therapy. Moreover, we found that ptNGS indeed exhibited higher sensitivity compared to mNGS. Our findings will be valuable to cases with comparable symptoms.

## Acknowledgments

We thank the Guizhou Bingyuan Youce Biotechnology Co. LTD.

## Author contributions

YL: Designed the work; QZ and YL: Acquired and analyzed data; All authors drafted, revised, and approved the manuscript.

**Conceptualization:** Qian Zhang, Ying Liu.

**Data curation:** Ying Liu.

**Formal analysis:** Ying Liu.

**Investigation:** Qian Zhang, Ying Liu.

**Methodology:** Junling Tao, Ying Liu.

**Project administration:** Ying Liu.

**Resources:** Ying Liu.

**Supervision:** Qian Zhang.

**Writing – original draft:** Qian Zhang, Jia Yuan, Wei Zhao, Weiwei Ouyang, Bowen Chen, Yehong Li, Junling Tao, Xianjun Chen, Guangsu Li, Zhendong Guo, Ying Liu.

**Writing – review & editing:** Ying Liu.
